# Oral health of patients suffering from end-stage solid organ insufficiency prior to solid organ re-transplantation: a retrospective case series study

**DOI:** 10.1186/s12903-021-01908-2

**Published:** 2021-10-24

**Authors:** Tobias Moest, Rainer Lutz, Arne Eric Jahn, Katharina Heller, Mario Schiffer, Werner Adler, James Deschner, Manuel Weber, Marco Rainer Kesting

**Affiliations:** 1 Department of Oral and Maxillofacial Surgery, University Hospital Erlangen, Glückstraße 11, 91054 Erlangen, Germany; 2 Department of Nephrology and Hypertension, University Hospital Erlangen, Ulmenweg 18, 91054 Erlangen, Germany; 3 Department of Medical Informatics, Biometry and Epidemiology (IMBE), University of Erlangen-Nuremberg, Waldstraße 6, 91054 Erlangen, Germany; 4 Department of Periodontology and Operative Dentistry, University of Mainz, Augustusplatz 2, 55131 Mainz, Germany

**Keywords:** Oral health, Organ transplantation, Immunosuppression

## Abstract

**Background:**

The oral health of organ transplanted patients before organ re-transplantation is largely unknown. This retrospective clinical study evaluates the necessity for intraoral surgical intervention and/or conservative treatment in candidates awaiting organ re-transplantation, both for graft failure and for reasons of another upcoming solid organ transplantation (renal or non-renal).

**Methods:**

From January 2015 to March 2020 n = 19 transplant recipients in evaluation on the waiting list for solid organ re-transplantation could be included in the retrospective case series study. Using clinical and radiological examinations, necessity for oral surgical or conservative dental treatment was evaluated. On the basis of anamnesis data, current kidney function, renal replacement treatment (RRT), and medication, a risk profile for several patient subgroups was created.

**Results:**

The clinical and radiological examinations showed a conservative and/or surgical treatment need in n = 13 cases (68.42%). In n = 7 cases (36.84%) surgical intervention was recommended due to residual root remnants (n = 5), unclear mucosal changes (n = 1), and periimplantitis (n = 1). In n = 16 recipients (84.2%) RRT (n = 15 hemodialysis; n = 1 peritoneal dialysis) had been performed. N = 14 recipients (73.68%) received immunosuppressants. In n = 1 patient (5.3%) displayed intraoral and n = 4 patients (21.1%) extraoral neoplasms due to drug-induced immunosuppression.

**Conclusions:**

Solid organ transplant recipients with renal failure present a complex treatment profile due to a double burden of uremia plus immunosuppressants. In cases of surgical treatment need a hospitalized setting is recommended, where potentially necessary follow-up care and close cooperation with disciplines of internal medicine is possible in order to avoid surgical and/or internal complications.

## Background

Solid organ transplantation is the lifesaving therapy for recipients with organ failure. Despite dialysis, kidney transplantation remains the gold standard for renal replacement treatment, prolonging life expectancy and quality of life [1]. Acute allograft rejection is a major cause of allograft dysfunction even with maximal antirejection therapy. For recipients who recover, acute rejection episodes can have a negative impact on long-term graft survival [2, 3]. Acute rejection is a major predictor for graft loss after the first year posttransplant [2]. There has been a dramatic reduction in the incidence of acute rejection due to the introduction of potent immunosuppressive drugs in the past 3 decades. However, optimizing immunosuppression to both prevent allograft rejection and minimize drug toxicity, new-onset diabetes, infection, and malignancy remains challenging [4, 5]. Risk factors for the development of acute rejection include pre-sensitization, presence of donor-specific antibodies, human leukocyte antigen (HLA) mismatches, pediatric recipient, African-American ethnicity, and delayed graft function [6]. In addition, patients with a previous episode of rejection, those receiving a second or greater transplant, and those with medication nonadherence are at increased risk for acute rejection [7, 8]. The acute rejection takes place within the first days or weeks after transplantation. It is caused by acute antibody production or acute T-cell activation [9]. Chronic transplant rejection can usually be observed more than 3 months after transplantation due to chronic antibody induction or chronic T-cell activation [10]. Because of the introduction of more advanced and potent immunosuppressive drugs, the success rate of organ transplants is continually improving, consequently lowering the incidence of organ rejection within the first year to approximately 7.9% [10]. Possible factors contributing to organ rejection may include prior sensitization of the recipient, the type of transplant donor (living vs. cadaveric), the duration of the ischemic phase after organ removal, HLA incompatibility, lack of compliance, previous organ rejection, or inadequate immunosuppression [11].

In this context, the simultaneous presence of chronic inflammation is discussed as a possible promoting factor for transplant failure since antigen-presenting cells could stimulate effector T-cells and the secretion of proinflammatory cytokines and lead to an up-regulation of major histocompatibility complex (MHC) -I and/or MHC-II molecules. In the oral cavity deeply destroyed teeth, apical inflammatory processes, and chronic periodontitis represent chronic inflammatory processes. It could be shown that there is a significant association between the local inflammatory situation due to chronic periodontitis and a systemic increase in inflammatory mediators, such as interleukin (IL) -1, IL-6, IL-8, and TNF-α [12, 13]. Il-6 represents a key role in periodontal pathogenesis, as it is associated with the stimulation of inflammatory acute phase proteins and the migration of immune cells. Il-6 also appears to be strongly associated with autoimmunity [14] and allotransplantation [15, 16]. For this reason, it is hypothesized that local additional inflammatory events lead to an induction of pro-inflammatory mediators, such as Il-6, in transplant patients [17–19]. These can become systemically effective and thus induce systemic inflammation [20], which can have a negative impact on graft survival. Although the scientific evidence for this is moderate, due to a small number of patients included in the study, oral inflammation seems to have an impact on systemic complications. This connection should not be overlooked by either dentists and oral and maxillofacial surgeons, or the transplantation team.

Past studies have already demonstrated impaired oral health in patients suffering from terminal renal insufficiency under dialysis and retrospective analyses have shown that the need for surgical rehabilitation of oral inflammatory foci is substantial before kidney transplantation [21]. In this context, the presence of intraoral inflammatory lesions, which may result in an induction of systemic inflammatory mediators in candidates awaiting re-transplantation, both for graft failure and for reasons of an upcoming other solid organ transplantation (renal or non-renal) is largely unknown.

The aim of this retrospective case series clinical study is to evaluate the necessity for surgical and conservative therapy in patients after previous solid organ transplantation and before renewed organ transplantation. Furthermore, internal medical and drug associated risk factors are to be identified and patient-oriented treatment recommendations formulated. As a working hypothesis, we assume an increased surgical and therapeutic need for oral inflammatory causes in patients presenting with organ dysfunction after previous organ transplantation.

## Methods

### Study characteristics

This retrospective clinical case series study was conducted at the Department of Oral and Maxillofacial Surgery of the University Hospital Erlangen, Germany, in cooperation with the Department of Nephrology and Hypertension, University Hospital Erlangen, Erlangen, Germany. The patients were admitted by the Department of Nephrology and Hypertension and routinely presented in the university outpatient clinic of the Department for Oral and Maxillofacial Surgery as part of the listing algorithm before organ transplantation for oral inspection and surgical/conservative treatment. Patients who were in compliance with our inclusion and exclusion criteria in the period from January 2015 to March 2020 were taken into account in this retrospective analysis.

This study was approved by the Ethics Committee of Friedrich-Alexander-University Erlangen-Nuremberg (Petition No. 450_20Bc). All procedures performed in studies involving human participants were in accordance with the ethical standards of the institutional and/or national research committee and with the 1964 Helsinki declaration and its later amendments or comparable ethical standards.

### Inclusion and exclusion criteria

Patients, regardless of age, previously transplanted organ, and dentition/number of residual teeth, who were referred to the Department of Oral and Maxillofacial Surgery of the University Hospital Erlangen by the Department of Nephrology and Hypertension between January 2015 and March 2020 for consultation and for intraoral/extraoral status evaluation prior to solid organ re-transplantation were included in the study.

Patients who refused a radiological or clinical assessment or whose transplantation- and dialysis dates was not complete were excluded.

According to the inclusion and exclusion criteria n = 19 patients aged between 19 and 79 years (mean: 51.95 ± 17.83 years, median: 54 years) were included in the study.

### Outcome variables

The primary outcome variable of the study was the quantification of the necessity for surgical treatment of inflammatory intraoral focuses prior to organ re-transplantation.

The secondary outcome variable is the evaluation of the causes of kidney transplant failure, as well as documentation of intraoral (gingiva proliferation) and extraoral neoplasms induced by immunosuppressive medications.

### Assessment of extraoral and intraoral status

All study participants were examined via a standardized procedure by an oral and/or maxillofacial surgeon. The clinical examination included the standardized extraoral inspection of the skin of the head and neck regions as well as an intraoral examination. Oral health was determined using the DMFT index method. Wisdom teeth were not taken into account. Clinically visible teeth were assessed for loosening and percussion sensitivity. The clinical examination includes the inspection of the entire oral mucosa focusing on inhomogeneities, swelling, bleeding, reddening, and neoplasm. The examination was completed by a standardized panoramic X-ray examination by performing an orthopantomogram (Sirona Orthophos XG; Sirona Dental Systems GmbH, Bensheim, Germany). The radiological analysis takes into account the assessment of teeth, roots or root remains, or the periodontium as well as intraosseous or peri-dental lesions such as cysts or other osteolysis. In addition, as far as possible, an assessment of the maxillary sinus was carried out.

### Demographic and clinical data

Information regarding age, gender, basic diseases, and permanent medications, as well as the abuse of nicotine, alcohol, and/or drugs, was collected via a standardized questionnaire. The specific immunosuppressant medication and/or the type of immunosuppressant, as well as transplantation-specific data (date of transplantation, living/cadaveric donation) and dialysis-specific data (date of re-dialysis after organ transplantation) were obtained.

### Determination of treatment need

The decision for surgical therapy was based on the analysis of the radiological and clinical findings. Apical processes after unsuccessful root canal treatment, other inflammatory bone changes, non-sustainable teeth due to strong loosening and bone loss, deep carious destruction of a tooth or residual root residues, or fractured teeth were defined as unsustainable.

A conservative approach was recommended for teeth that had treatable carious lesions, whose percussion was positively paired with negative vitality, or for teeth with initial periodontitis.

### Statistical analysis

Due to the small sample size in our study, we did not perform statistical testing. For categorical variables, proportions are reported, and for metric variables, mean values and standard deviations are given. All statistical analysis was done using R V3.6.3 [22].

## Results

### Characterization of study population

Of the included patients n = 10 (52.6%) cases were female. In n = 18 cases (94.7%) patients were referred to the Department of Oral and Maxillofacial Surgery after previous kidney transplantation. In these cases, the mean time interval that the transplanted kidney had been in situ was 15.61 + 9.77 years (median: 14.00 years). One patient (n = 1; 5.3%) had undergone a liver transplantation and subsequently developed renal insufficiency. In n = 1 patient (5.3%) a kidney transplantation had been performed before a liver transplantation was planned. In n = 1 patient (5.3%) a combined heart and kidney transplantation followed by a kidney re-transplantation had previously been performed. In a total of n = 5 cases (26.3%), kidney(s) had already been re-transplanted. In n = 6 cases (31.6%) kidney transplants were generated from living donors. At the time of consultative presentation, in n = 15 cases (78.9%) patients received hemodialysis, and n = 1 patient (5.3%) received peritoneal dialysis. In n = 3 cases (15.8%) no dialysis was performed. In n = 12 patients (63.2%) the previous transplanted organs were in situ.

Drug-induced immunosuppression was performed in n = 14 cases (73.7%): in n = 13 cases (68.4%) due to previous kidney transplantation and n = 1 case (5.3%) with simultaneous heart transplantation and in n = 1 case (5.3%) due to a previous liver transplantation. Two cases (n = 2; 10.5%) were under gradual reduction of glucocorticoids after kidney transplant removal. Three patients (n = 3; 15.8%) received three different types of immunosuppressive medications, n = 5 patients (26.3%) double immunosuppressive, and n = 6 patients (31.6%) single immunosuppressive therapy. Several data are presented in Table 1.

**Table 1:** Demographic, transplantation, dialysis data and list of drugs which were taken for immunosuppression by the included patients

Patients	Age	Previous transplantation(s)	Donors	Actual organ insufficiency	Transplanted organs in situ	Dialysis	Immunosupressive medication	Number of immunosuppressive medications	Type and combination of immunosuppressive medications
1	35	Kidney	Living	Kidney	No	Hemodialysis	No	–	–
2	54	Liver	Cadaveric	Kidney	Yes	No dialysis	Yes	3	Tacrolimus, Mycophenolat, Glucocorticoides
3	79	Kidney	Living	Kidney	Yes	Hemodialysis	Yes	1	Tacrolimus
4	48	Kidney	Cadaveric	Kidney	No	Hemodialysis	No	–	–
5	29	Kidney	Living	Kidney	No	Hemodialysis	No	–	–
6	57	Kidney	Living	Liver	Yes	No dialysis	Yes	2	Sirolimus, Glucocorticoides
7	25	Kidney	Cadaveric	Kidney	No	Hemodialysis	Yes	1	Glucocorticoides
8	61	Kidney, Heart	Cadaveric	Kidney	Yes	Hemodialysis	Yes	3	Tacrolimus, Mycophenolat, Glucocorticoides
9	56	Kidney	Cadaveric	Kidney	No	Hemodialysis	Yes	1	Glucocorticoides
10	78	Kidney	Cadaveric	Kidney	Yes	Hemodialysis	Yes	1	Glucocorticoides
11	64	Kidney	Cadaveric	Kidney	Yes	No dialysis	Yes	2	Azathioprin, Glucocorticoides
12	46	Kidney	Living	Kidney	Yes	Hemodialysis	Yes	1	Tacrolimus
13	71	Kidney	Cadaveric	Kidney	Yes	Hemodialysis	Yes	2	Cyclosporin, Glucocorticoides
14	51	Kidney	Cadaveric	Kidney	Yes	Hemodialysis	Yes	1	Cyclosporin
15	75	Kidney	Cadaveric	Kidney	Yes	Hemodialysis	Yes	2	Cyclosporin, Glucocorticoides
16	40	Kidney	Cadaveric	Kidney	No	Hemodialysis	No	–	–
17	19	Kidney	Living	Kidney	Yes	Peritoneal Dialysis	Yes	2	Tacrolimus, Glucocorticoides
18	62	Kidney	Cadaveric	Kidney	Yes	Hemodialysis	Yes	3	Belatacept, Mycophenolate, Glucocorticoides
19	37	Kidney	Cadaveric	Kidney	No	Hemodialysis	No	–	–

**Fig. 1:**
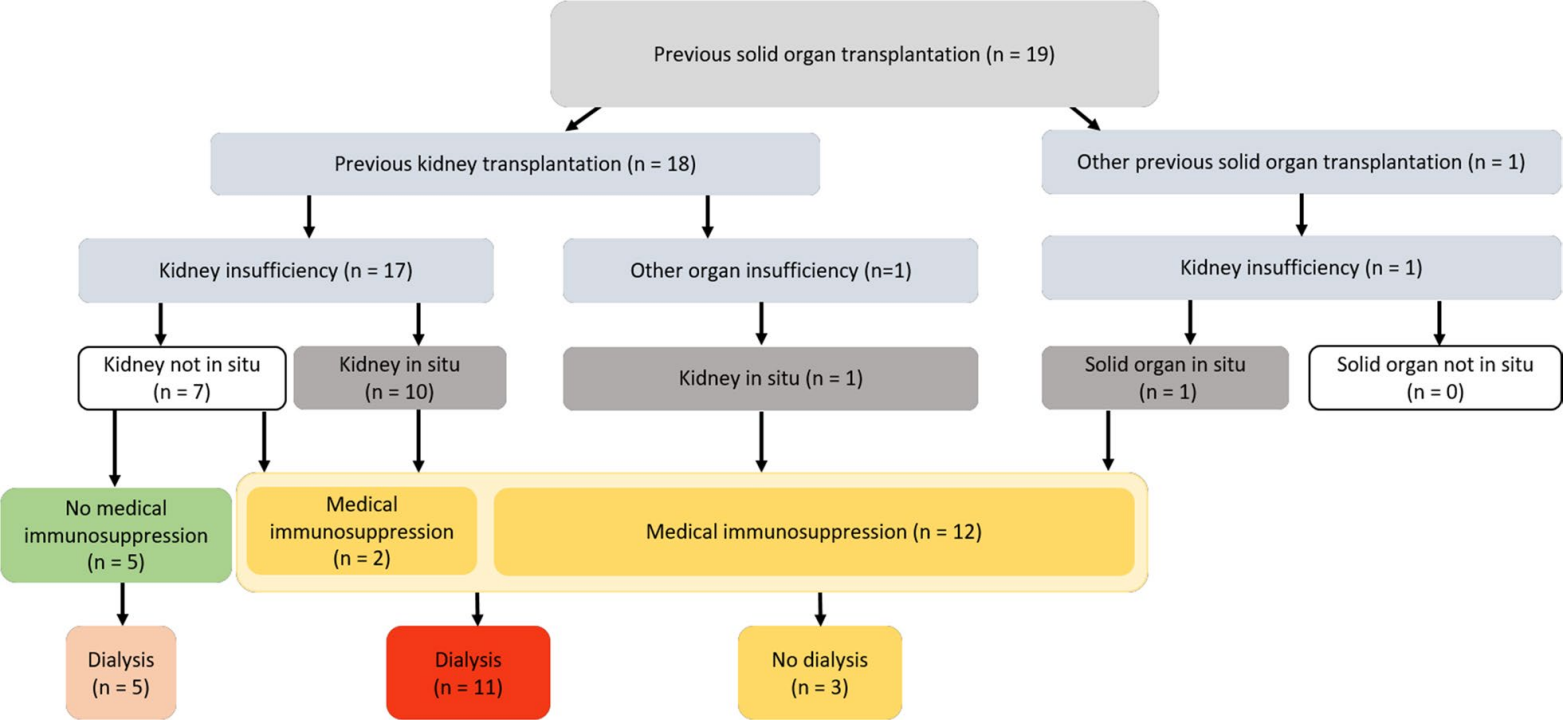
Categorization of study population according to risk factors (drug-induced immunosuppression, dialysis). Category I (red; very high risk of complications), category II (bright red; high risk of complications), and category III (yellow, risk of complications)

Based on the aforementioned risk factors (immunosuppression and dialysis), the study collective can be classified into different groups based on their transplantation history (Fig. 1).

### Reasons for kidney transplant insufficiency

In n = 6 cases (31.6%) the transplanted kidneys were insufficient due to chronic allogenic nephropathy. In n = 5 cases (26.3%) infections of the urinary tract led to chronic graft loss. In n = 1 case (5.3%) each, transplant dysfunction was the result of either vascular rejection, discontinuation of immunosuppressive medication, desiccosis, lack of volume during surgical intervention, relapse of an IgA nephropathy, or an infectious-toxic genesis.

### Harmful use of substances

In n = 8 cases (42.1%) no harmful use of substances could be documented. In n = 5 cases (26.3%) alcohol was consumed on occasion. In n = 1 case (5.3%) nicotine abuse and a harmful use of analgetic drugs were present. In n = 5 cases (26.3%) patients had formerly abused nicotine/alcohol.

### Oral status with conservative and surgical treatment need

The clinical and radiological examinations showed that in n = 13 cases (68.4%), conservative and/or surgical treatment need existed. In n = 10 cases (52.6%) there was indication for conservative treatment (periodontitis treatment, root channel treatment, and root channel revision filling therapy). In n = 7 cases (36.8%) surgical intervention was recommended due to residual root remnants (n = 5), unclear alterations of the oral mucosa (n = 1), and periimplantitis (n = 1; 50.0%). The number of teeth recommended for removal ranged from one to eight teeth. In n = 4 cases (21.1%) a conservative as well as surgical treatment need was present.

**Fig. 2:**
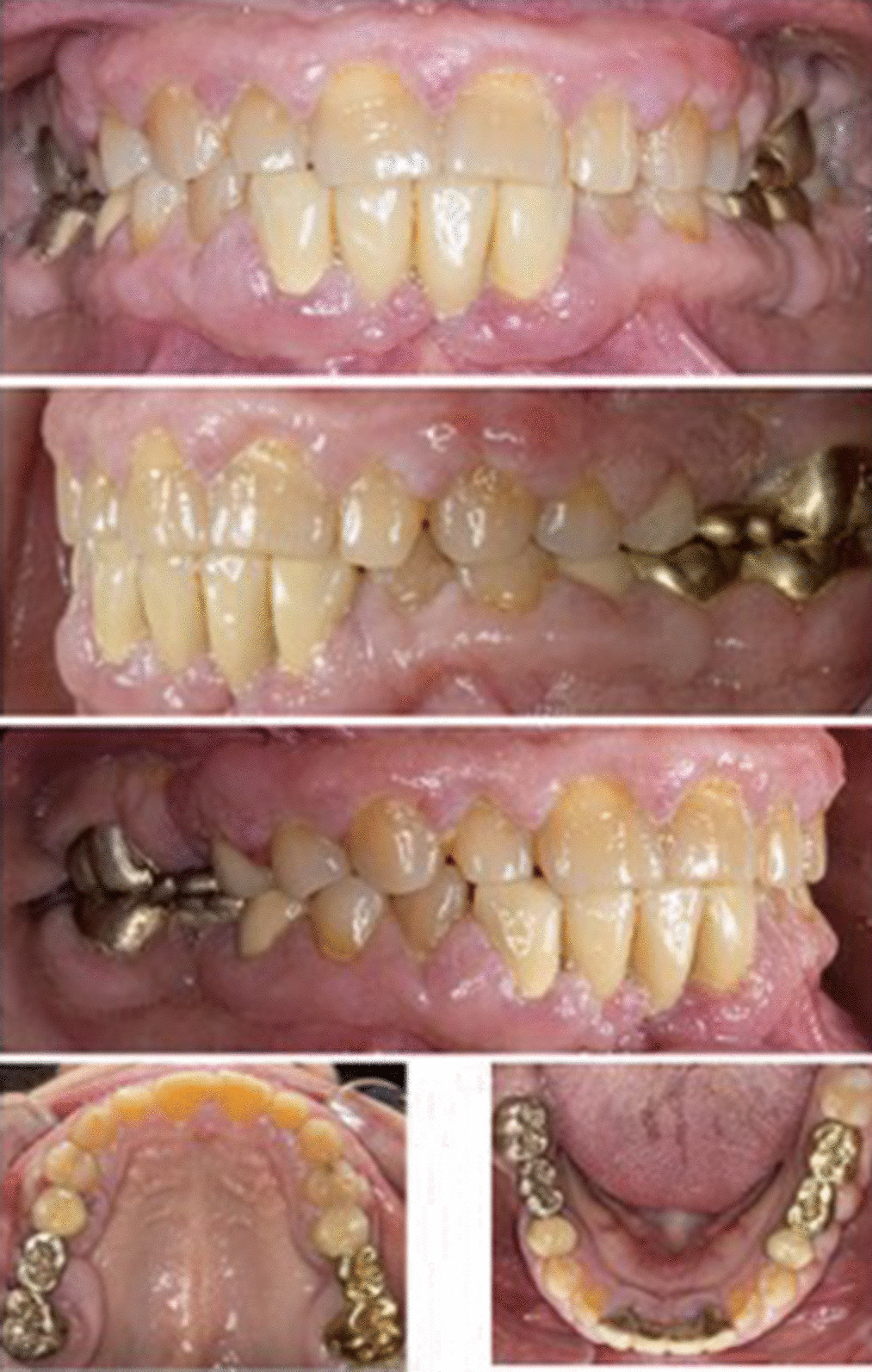
Clinical case presentation of a female patient with a moderate gingiva hyperplasia due to cyclosporin medication

In n = 1 (5.3%) patient, gingival hyperplasia in the upper and lower jaw could be detected (Fig. 2). In this patient, immunosuppression had been achieved by cyclosporin since 1994. In n = 4 cases (21.1%) cutaneous neoplasm, in n = 3 cases (15.%) basalioma, in n = 1 case (5.3%) facial angiofibromas of tuberous sclerosis, and in n = 1 case (5.3%) squamous cell carcinoma in situ (Bowen’s disease) in the head and neck area could be evaluated. In one case basalioma and Bowen’s disease were documented at the same time. Several data are presented in Table 2.

**Table 2:** Distribution of conservative and surgical treatment need as well as intraoral and extraoral immunosuppression-induced findings

Patients	Conservative treatment need	Surgical treatment need	Intraoral immunosuppression-induced finding	Extraoral immunosuppression-induced finding
1	No	No	–	–
2	Yes	No	–	–
3	Yes	Yes	–	Basalioma, Squamous cell carcinoma in situ (Bowens’s disease)
4	Yes	Yes	–	–
5	Yes	No	–	Facial angiofibromas of tuberous sclerosis
6	Yes	No	–	–
7	No	No	–	–
8	Yes	Yes	–	–
9	Yes	No	–	–
10	Yes	No	–	Basalioma
11	Yes	Yes	–	–
12	No	Yes	–	Basalioma
13	No	No	–	–
14	No	No	Gingiva hyperplasia	–
15	No	Yes	–	–
16	No	Yes	–	–
17	No	No	–	–
18	No	No	–	–
19	Yes	No	–	–

## Discussion

The aim of the study was to determine the conservative and surgical treatment need in transplant recipients awaiting re-transplantation of a renal or non-renal solid organ either under dialysis or under immunosuppressants or both. The retrospective analysis shows a treatment requirement of 68.4%, with 36.8% of all cases requiring surgical treatment. Taking into account the low absolute numbers (n = 19) of patients that could be observed within the relatively long interval of over 5 years, it must be noted that the examined patient collective is small and they thus represent a rarity in oral or oral and maxillofacial practice.

Nonetheless, at the time of this study, no scientific literature addressing this patient collective existed, so a characterization of these patients with a description of specific risk factors and treatment recommendations is essential.

The successful treatment of inflammatory lesions represents a basic requirement before organ (re-) transplantation, in order to prevent a possible exacerbation of inflammation, with the risk of development of life-threatening abscesses during the drug-induced immunosuppression for organ transplantation. In the phase after organ transplantation, the non-inflammatory status must be maintained, since, in addition to a dreaded local spread of inflammation, a possible interruption of the drug-induced immunosuppression might become necessary in order to treat the abscess and ensure the survival of the patient. Any reduction of immunosuppression leads to an increase in immune competence, possibly resulting in transplant rejection and thus also endangering the patient's survival. In addition, persistent local inflammatory lesions can lead to an increased level of inflammatory mediators, which can have the systemic effect of organ rejection [15, 16]. In this context, the inflammatory mediator Il- 6, which is raised in patients with chronic organ rejection but is also overexpressed in intraoral inflammatory events, seems to have special importance [20].

For these reasons, the treatment of intraoral inflammatory events before and after organ transplantation under drug-induced immunosuppression is essential to guarantee long-term transplant success.

In our patient group, surgical treatment need was necessary in more than 36% of all cases, indicating the need for complex treatment planning, when taking into account the patient-specific anamnesis and permanent medications. In the context of treatment planning, all existing risk factors (Fig. 1) should be taken into account and utilized to categorize organ transplanted patients into different risk profiles.

In patients who had already received a previous organ transplant, an essential question is whether the transplanted organ is still in situ. If this is the case, drug immunosuppression is obligatory. Due to the risk of wound healing disorders, we recommend the administration of preoperative intravenous antibiotics and their continued administration over several days during a stay in hospital. Furthermore, a parenteral diet by application of a nasogastric tube in order to minimize mechanical irritation of the wound is recommended. Intraoperatively, sharp bone edges should be removed and a plastic closure of the wound must be achieved in a minimally invasive procedure. Our data shows that in some patients, other solid organs (e.g., in one case a liver transplantation had been performed) were transplanted before the planned kidney transplant. In these cases, special attention must be paid to the transplanted organ, since, even in cases of successful transplantations, the functional capacity of the transplanted organ system may be below the normal range. Therefore, a consultation with the respective internal specialist is highly recommended before the surgical intervention and the accompanying application of drugs (local anesthetics, analgetics, and antibiotics). However, in addition it must be taken into account that the application of immunosuppressive drugs in some patients can still be necessary after transplant explantation, in order to achieve a gradual weaning of recently high-dose corticosteroid applications.

For competent treatment planning, knowledge of dialysis schedules is fundamental as well. As a rule, dialysis is performed three times per week. The necessary surgical intervention should be carried out on dialysis-free days to reduce the risk of post-surgical bleeding due to dialysis-related anticoagulation. In situations of several extractions or the need for osteotomies, hospitalization can be useful again. If a multi-day hospitalization is indicated, e.g., due to other drug-based risk factors (immunosuppressive drugs, antiresorptive medication), the preoperative organization of the dialysis protocol with continuation of the dialysis during the hospital stay in a dialysis center of the specific facility is indicated. The dialysis protocol has to be organized before performing the surgical intervention.

On the basis of these risk factors, the organ transplanted patients with indication for organ re-transplantation presented to our department could be categorized into category I (drug-induced immunosuppression + dialysis; red) with very high risk of complications, category II (no drug-induced immunosuppression + dialysis; bright red) with high risk of complications, and category III (drug-induced immunosuppression + no dialysis; yellow) with risk of complications.

In addition to the aforementioned internal risk factors, the influence of drug-induced immunosuppression on the development of intraoral and cutaneous neoplasms must also be taken into account, since the development of skin tumors is significantly increased [23–25] and characterized by a more aggressive behavior [26]. In this context, in more than 20% of the included patients, lesions of the outer skin (basaliomas or Bowen carcinomas) were documented. One patient showed cyclosporin associated gingival hyperplasia.

The immunosuppressed patient must be made aware of this fact and given competent and regular tumor aftercare.

Overall, organ-transplanted renally or other solid organ insufficient patients represent a complex patient population, which is characterized by an extremely high internal, surgical, and dental risk profile. In order to achieve maximum therapeutic success, close communication must take place between the respective transplant center and doctors of the surgical discipline. From the point of view of oral medicine, it is important to treat these patients as holistically as possible in an interdisciplinary way due to the complex therapeutic demands in cases of surgical and conserving therapy requirements. The organization of a proper regime of care for this group of patients, due to its complexity and multifaceted nature, should best be managed by a team of interdisciplinary specialists within an appropriate clinical setting.

## Conclusions

Solid organ transplant recipients with renal failure present a complex treatment profile due to a double burden of uremia plus immunosuppressants. Surgical procedures should be performed in a hospitalized setting, where potentially necessary follow-up care and close cooperation with disciplines of internal medicine is possible.

## Data Availability

The data and materials collected in this research are available from the corresponding author when requested reasonably.

## Abbreviations

RRT: Renal replacement treatment
HLA: Human leukocyte antigen
MHC: Major histocompatibility complex
IL: Interleukin
TNF-α: Tumor necrosis factor alpha
DMFT Index: Decayed-missing-filled teeth index
N: Number
